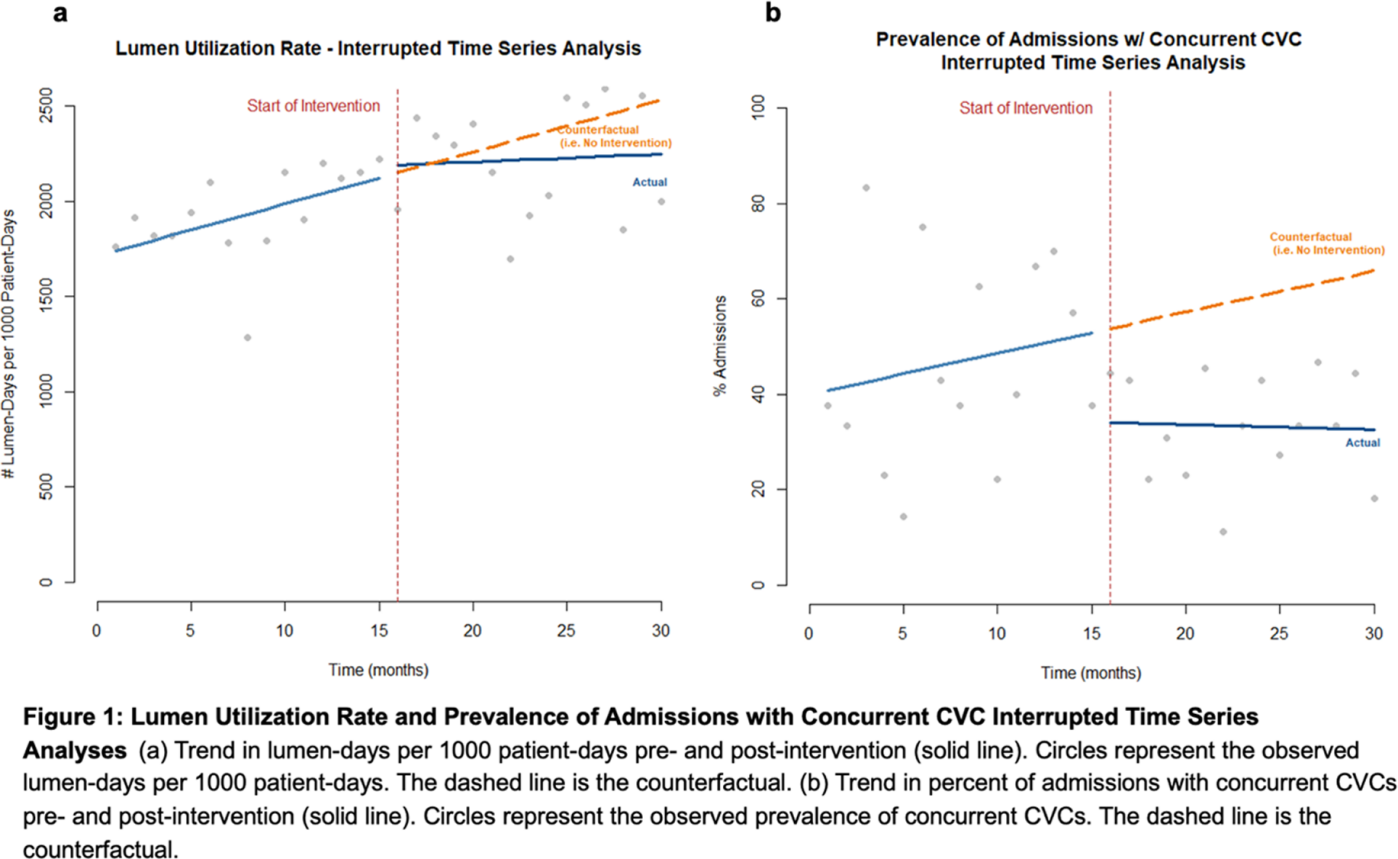# 165 Enhancing Capacity to Properly Perform Infection Prevention and Control Practices Through a Multimodal Approach to Building Knowledge

**DOI:** 10.1017/ash.2026.10566

**Published:** 2026-06-23

**Authors:** Yokabed Ermias, Sarah Blaine, Lauren Quinn, Julianne Gent, Lindsey Gottlieb, Bashar Staitieh, Jessica Howard-Anderson

**Affiliations:** 1 Emory University; 2 Emory Healthcare; 3 Emory University School of Medicine

## Abstract

**Background:** Patients in the intensive care unit (ICU) often require two concurrent central venous catheters (CVCs) – a temporary CVC for medication administration and a hemodialysis (HD) catheter (HDC). Having concurrent CVCs increases the risk of central line-associated bloodstream infections (CLABSIs). Triple-lumen HDCs have an additional lumen, which may reduce the need for concurrent CVCs as compared to double-lumen HDCs. We evaluated CVC utilization and prevalence of concurrent CVCs in patients with HDCs in an academic ICU after implementing triple-lumen HDC use. **Methods:** We analyzed data from patients with newly placed HDCs, admitted to a 20-bed medical ICU in an academic hospital in Atlanta, GA from November 2022-January 2025. Triple-lumen HDCs were introduced February 2024. Data were obtained from the electronic health records. Concurrent CVC was defined as having a newly placed HDC and any other simultaneous CVC for at least one calendar day during the ICU admission. Central line-days were defined as number of CVCs in place per 1000 patient-days and lumen-days were defined as number of lumens in place per 1000 patient-days. We analyzed CVC and lumen utilization and prevalence of concurrent CVCs after the intervention by interrupted time series analysis (ITS). ITS with Poisson regression determined if there was a decrease in lumen-days or admissions with concurrent CVCs in the post-intervention vs. pre-intervention time period. An exploratory analysis compared the number of CLABSIs (as defined by NHSN) pre- and post-intervention. **Results:** **Conclusion:** After the introduction of triple-lumen HDCs to an academic medical ICU, the total prevalence of patients on HD requiring concurrent CVCs decreased, however our sample size was too small to demonstrate statistical significance by ITS. Decreasing the need for concurrent CVCs may reduce CLABSIs in this ICU but additional time post-intervention is needed.

**Figure:**